# Book Review

**Published:** 1940

**Authors:** 


### Reviews of Books

**Landmarks and Surface Marking of the Human Body.** Eighth Edition. By L. B. Rawling, M.B., B.C., F.R.C.S. Pp. viii., 98. Illustrated. London: H. K. Lewis & Co. Ltd. 1940. Price 8s. 6d.—This book has been well known to generations of students as the standard work on the subject with which it deals, to which statement ample testimony is forthcoming in the fact that this is the eighth edition, and that the first appeared as long ago as 1904. While the facts it contains, and the way they are set out, remain as of old, there is one important alteration in the shape of the adoption of the British Revision of the B.N.A. Terminology. This step will probably appeal to the majority of readers, although there will no doubt still be a not inconsiderable minority who would have preferred to make no change. As to the subject-matter, the book contains, as before, all that need be known of the subject, set out in the simplest way and with adequate detail. The illustrations are clear and the index is complete. No student need fear to face his examiners if he has a knowledge of everything set out here; to learn the subject thoroughly he must not be tempted to let good letterpress and clear illustrations be anything more than valuable if it is supplemented by sufficient practice on the living subject: he will find this small book of untold value.

**Cunningham's Manual of Practical Anatomy.** Three volumes. Tenth Edition. Edited by J. C. Brash and E. B. Jamieson. Pp. x., 535. Illustrated. London: Oxford University Press (Humphrey Milford). 1940. Price 15s. each volume.—The general character of this well-known dissector's guide is unaltered, but several pieces of descriptive detail have well been omitted. Surface anatomy, lymph-drainage, relation of structure to function and applied anatomy have all received more attention than in early editions. New radiographs are added and there is an excellent general introduction. Instead of the unsatisfactory B.N.A., the Birmingham nomenclature is used, as accepted now by all British teachers. The binding, printing, illustrations and instructions are excellent. With so many revisions in each edition, it is difficult to see how any student can go wrong in following these three admirable, if rather portly, volumes.

**Insect Pests.** Wm. Clunie Harvey, M.D., D.P.H., and Harry Hill, A.M.I.S.E. Pp. ix., 292. Illustrated. London: H. K. Lewis & Co. Ltd. 1940. Price 10s. 6d.—Of all the irritating nuisances with which a public health department is called upon to deal, vermin infestation is at once the most troublesome and perhaps the least satisfactory: for complete eradication is seldom achieved without most exacting and expensive measures having to be carried out. The appearance of this book will be welcomed. The authors have set out the essential principles and the proper methods of practice which should do much towards eliminating this menace to health and comfort. The arrangement of the subject-matter is good. The first part gives lucid details of the appearance, characteristics, and life-history of all the principal insect pests. The second deals with principles and practice of disinfestation and describes elimination of harbourage, gaseous fumigants, insecticides, and methods of control. A chapter is devoted to legislative measures, including ship inspection and disinfestation. The book can be recommended to public health officials, housing managers, and people having control of dwelling-houses, though some mention might have been made of furniture mites. It is well illustrated and, being furnished with a complete index, meets a definite need for those whose duty is the control of insect pests.

**Applied Physiology.** Seventh Edition. By SAMSON WRIGHT, M.D., F.R.C.P. Pp. xl., 787. Illustrated. London: Oxford University Press (Humphrey Milford). 1940. Price 25s.—Yet another edition of this well-known book, the seventh in fourteen years, has appeared. The revision seems to have been carried out with great thoroughness, and the author is to be congratulated on his success in bringing the various chapters up to date. It is invidious to attempt to single out the best parts of a book which sets a high standard throughout, but one may point particularly to the sections on the circulation; the pages on hypertension and electrocardiography call for special mention. The seventh edition is a hundred pages longer than its predecessor. While this is no disadvantage in itself, the increased weight of the book together with the new slippery binding cover does not make for comfortable holding. This is a small criticism, however, and one is glad to note that the publishers' usual standard is fully maintained. The book will continue to be popular with medical students just before and during their clinical work, as well as with practitioners who want to obtain in a concise form current information on the applications of Physiology to Medicine.

**Illustrations of Bandaging and First Aid.** Compiled by LOIS OAKES, S.R.N., D.N. Pp. viii., 248. Illustrated. Edinburgh: E. & S. Livingstone. 1940. Price 6s.—The book is extremely well produced and the photographs excellent. It is pointed out in the preface that correct positions of the operators are not always shown, nor is support of the limb always shown, *e.g.* the foot, on page 63. The roller bandage to eye, page 147, would not be considered good by many ophthalmic surgeons, who object to an upward bandage as likely to produce pressure. The book is especially useful as it includes first-aid methods, and should prove particularly valuable to instructors of practical classes.

**Hæmorrhoids and Their Treatment.** By KASPAR BLOND, M.D., translated from the German by E. STANLEY LEE, M.S., F.R.C.S. Pp. vii., 140. Illustrated. Bristol: John Wright & Sons Ltd. 1940. Price 15s.—Injection therapy is now a well-recognized and firmly established method of dealing with certain types of hæmorrhoids and other ano-rectal lesions. This book reveals the author's enthusiasm for this form of treatment, but he is careful to realize that operation must still be the method of choice in some cases. It should prove invaluable to those who favour the ambulatory treatment of rectal varicose disease, for the section dealing with the technique of injection is well written and gives a full and detailed account of the methods employed. The main interest in this book, however, lies in the author's attempt to find a satisfactory explanation for the causation of hæmorrhoids. His arguments, stressing the close relationship between ano-rectal varices and the portal circulation are convincing, and should do much to clarify our present knowledge of the pathology and treatment of this condition. The book is attractively produced, the illustrations being excellent.

**Manson's Tropical Diseases.**—Eleventh Edition. By PHILIP H. MANSON-BAHR, C.M.G., D.S.O., M.D., F.R.C.P. Pp. xvi., 1083. Illustrated. London: Cassell & Co. Ltd. 1940. Price 35s.—This book requires little in the way of commendation. It is now in its eleventh edition revised, and although Sir Patrick Manson in the *envoi* to the first edition in May, 1898, stated that it made no pretension to being anything more than an introduction, and was "in no sense put forward as a complete treatise," the eleventh edition certainly contains all that anyone but the most advanced practitioner could require. Although the chapter on Technique is omitted, this volume contains 150 pages more than the ninth edition, which is the last volume of which the reviewer has a copy. There are excellent chapters added upon Life in the Tropics, the Passing of Candidates, the Problems of Nutrition, etc. Much ancient history has been pruned away to make room for newer work. Wherever one turns in this excellent volume one finds readily accessible and accurate data upon all subjects. Recent work, such as that on the etiology of yellow fever and the treatment of pellagra, to quote only two subjects, is admirably presented. Each edition makes this time-honoured friend a more indispensable *vade mecum* for all practising medicine in warmer climates.

**Diseases of the Nervous System.** By F. M. R. Walshe, O.B.E., M.D., D.Sc., F.R.C.P. Pp. xiii., 288. Illustrated. Edinburgh: E. & S. Livingstone. 1940. Price 12s. 6d.—Most text-books of neurology devote almost as much space to clinical curiosities as they do to the common disorders, and in addition credit the reader with a knowledge of neuro-physiology that only rarely exists. Dr. Walshe's book can be recommended primarily for the fact that he has purposely avoided these two pit-falls. "Curiosities" are not even mentioned, but the diseases and syndromes surveyed are dealt with clearly and from the clinical approach. The London School of Neurology is a credit to British medicine, and this book in turn is a credit to that school.

**Psycho-Analysis.** By EDWARD GLOVER. Pp. viii., 139. Illustrated. London: John Bale Medical Publications Ltd. 1939. Price 10s. 6d. **An Investigation of the Technique of Psycho-Analysis.** By EDWARD GLOVER, M.D., with MARJORIE BRIERLEY, M.B., B.Sc. Pp. ix., 188. London: Ballière, Tindall & Cox. 1940. Price 10s. 6d.—Recent years have witnessed an ever-developing interest in psychological medicine, and there has been a corresponding spate of books on the subject. These two recent additions to the bibliography are by one of the leading exponents of the subject in this country, the head of the Freudian school in this country. The first is a useful survey of the subject and can be recommended to those who desire a fairly detailed exposition. The author recognizes the limitations of this method of treatment, which are certainly very real, but at the same time he presents a well-balanced plea for adequate treatment of the treatable cases. The second book is not of general interest and is devoted to the minutiæ of treatment. The author uses the questionnaire technique applied to his colleagues, but the results are colourless and only occasionally helpful.

**Neurosyphilis.** By C. WORSTER-DROUGHT, M.D., F.R.C.P. Pp. xiv., 241. Illustrated. London: John Bale, Sons & Staples Ltd. 1940. Price 10s. 6d.—It is always a pleasure to read a book that shows evidence of sound scholarship in the author, and Worster-Drought's neurosyphilis certainly falls into this category. He has covered the whole subject most competently and the references show his knowledge of good Continental and American literature. Although pathology and treatment are dealt with adequately, the book is devoted predominantly to the clinical aspect of the disease. This work can be recommended to all those who need a sound survey of the subject; it is too detailed for the medical student.

**The Diagnosis and Treatment of Diseases of the Peripheral Arteries.** Second Edition. By SAUL S. SAMUELS, M.D. Pp. xxiii., 372. Illustrated. New York: Oxford University Press. 1940. Price 31s. 6d.—The author has had what must be the unrivalled experience of studying upwards of 500 cases of angiitis obliterans and more than half his book is devoted to consideration of this disease. Of all the cases which he has seen, only five were in women and in only three cases was he compelled to resort to a major amputation. The author stresses the importance of early diagnosis and the advantages of the Pachon oscillometer. He has also elaborated a test for defective circulation in the feet, which is certainly little known in this country. When the legs are raised and the ankle voluntarily flexed and extended rapidly he states that blanching of the soles is observed when even a minor degree of vascular obstruction is present. He emphasizes the readiness with which a good collateral circulation is established, hence the importance of delaying amputation. He also stresses the dire effects of tobacco in this disease and how important it is that its use should be absolutely prohibited. Other vascular diseases are adequately discussed though with less originality than in the case of angiitis obliterans. The book as a whole is well printed, indexed and illustrated, and any medical man will discover in it much to interest him; the time taken to read it will be time well spent.

**A Textbook of Dietetics.** By L. S. P. DAVIDSON, M.D., F.R.C.P., F.R.S.E., and IAN A. ANDERSON, M.B., Ch.B. Pp. xviii., 324. London: Hamish Hamilton Medical Books. 1940. Price 10s. 6d.—Professor Davidson and Dr. Anderson have produced an invaluable text book of Dietetics. As Sir John Orr says in his " Foreword " : " Common sense advice on diet and the application of dietetic measures for the promotion of health and the treatment of disease are well within the competence of doctors properly instructed in the physiology of nutrition." The modern medical student carries far more physiology into the hospital wards than did his predecessor of forty years back. So, too, the modern practitioner continues to apply his knowledge of physiology in every branch of practice: and in dealing with problems of nutrition and diet he has a solid foundation of physiology upon which to build. A book such as this connects the physiology of nutrition in a fascinating way with the detailed guidance for feeding both in health and in disease. One chapter in particular is most impressive in the clear arguments brought forward to justify the changes that have taken place in recent years in the diet appropriate to renal and urinary tract diseases. The changes have been radical, but the physiological reasons for them are given in the most convincing and intelligible manner. The diet sheets constructed by Miss Mary E. Thomson are admirable in their simplicity, palatability and variety. This is a grand little book for practitioners.

**Tomography.** J. B. McDougall, M.D., F.R.C.P., F.R.S.E. Pp. ii., 73. Illustrated. London: H. K. Lewis & Co. Ltd. 1940. Price 21s.—"Tomography" is the name adopted in this country for a comparatively recently developed method of X-ray examination whereby different layers or sections of parts of the body can be projected and recorded on films by means of special apparatus. The title of this book is somewhat misleading. It is not a treatise on Tomography, a subject which already has an extensive literature and has daily widening applications, but rather an excellent explanatory atlas. The author's aim is to describe and illustrate the applications of this method mainly in the investigation of diseases of the chest, particularly tuberculosis, a subject in which he has evidently had much experience. There is a brief introductory section on the principles of Tomography with notes on the technique employed by the author using the "Sanitas" machine. The major part of the volume is made up of about 100 radiographs well reproduced, including plain pictures and tomographs, which convincingly illustrate the value of the method in selected cases. To the radiographs of each case are appended valuable short explanatory notes and comments. The author has included a few tomographs from the collection of the late Dr. E. W. Twining (one of the pioneers of the methods in this country) to illustrate its application to other parts of the body. Its fields of application are in fact widening daily, but the technical problems connected with the method are often more elaborate and difficult than the author suggests. At the end of the volume there is a summary and an extensive but incomplete bibliography. This book should be studied particularly by those physicians and surgeons who are concerned with diseases of the chest, in order to acquaint themselves with some of the possibilities of this specialized method of examination.

**A Handbook of Radiography.** By John A. Ross, M.R.C.S., L.R.C.P., D.M.R.E. Pp. viii., 126. Illustrated. London: H. K. Lewis & Co., Ltd. 1940. Price 7s. 6d.—This is a handbook for students training to take the Membership Examination of the Society of Radiographers. It is not intended to be a text-book and blank pages are provided for notes. A large part of the book is devoted to the main positions generally employed. These are illustrated by simple line drawings. Special examinations are described briefly, and there is a useful chapter on localization of foreign bodies. Many useful formulæ are given. The general style is clear and brief.

**Surgery of the Alimentary Tract.** By SIR H. DEVINE, M.S., F.R.A.C.S., F.A.C.S. Pp. xi., 1046. Illustrated. Bristol: John Wright & Sons Ltd. 1940. Price 63s.—This book is probably the most important contribution that Australia has made to surgical literature. It is a record of personal experience on a large scale, and deals unusually fully with the diagnosis of important abdominal diseases when, as so often happens, the clinical picture diverges from that given in ordinary text-books, and disastrous mistakes are likely to be made. The author makes a valuable distinction between the dyspepsias troubling the patient during *filling* of the stomach and those occurring during *emptying*. His remarks on treatment and descriptions of operative technique are very good, and excellently illustrated. The most important of his recommendations is his method of de-functioning the colon as a preliminary to resection for growths or diverticulitis. He accepts the modern teaching that morphia increases the movements of the small intestine and averts paralytic distension of the abdomen. Every surgeon who treats abdominal cases should read this book with care, and though he may not always agree with all the opinions expressed with regard to treatment, he will find them well worthy of consideration. For the practitioner the chapters on Diagnosis are valuable, and may enable him to avoid missing the atypical case that is so apt to be recognized too late, such as pelvic appendicitis, appendicitis in men past sixty, or cancer of the colon without obstruction symptoms. In Australia, apparently, the diagnosis of gall-bladder disease is a good deal complicated by the occurrence of hydatid cysts rupturing into the bile passages.

**Pye's Surgical Handicraft.** Twelfth Edition. By HAMILTON BAILEY, F.R.C.S. Pp. xii., 595. Illustrated. Bristol: John Wright & Sons Ltd. 1940. Price 21s.—This well-known work is addressed mainly to house surgeons and surgical dressers, but in its new form there is included a considerable amount of exceedingly useful and often required information. Some thirty-eight well-known authorities have contributed sections on different surgical topics. The work is edited by Mr. Hamilton Bailey, who, as is his usual custom, has illustrated it freely with excellent diagrams and photographs. The book is well produced on good paper and the type is easy to read. A few more references at the ends of the sections might have been a small but useful addition. There are one or two short chapters at the end of particular usefulness to house surgeons on hospital administration, certification of death, and medico-legal reports. There are many useful matters included which are somehow often missed in the curriculum. An excellent account is given of modern plaster technique, and the immediate treatment of many of the commoner fractures. The index is good and very complete. The book can be recommended with confidence to any new houseman.

**Diseases of the Urethra and Penis.** By E. D'ARCY MCCREA, M.D., M.Ch., F.R.C.S.I., F.R.C.S. Pp. vii., 306. Illustrated. Bristol: John Wright & Sons Ltd. 1940. Price 21s.—This is a detailed monograph which covers thoroughly all the commoner and many of the rare conditions found in the urethra and penis. It is a useful reference work, and it is profusely illustrated with good clear diagrams. At the end of each chapter is a good list of references to other books and papers. The operational diagrams and details are simple and easy to follow. The text is clear and straightforward. The whole work is well produced and printed in good type on high-quality paper. The index is full and well arranged. It is a book to be recommended to anyone seeking special information in this class of case.

**Surgery of the Hand.** By R. M. HANDFIELD-JONES, M.C., M.S., F.R.C.S. Pp. viii., 140. Illustrated. Edinburgh : E. & S. Livingstone. 1940. Price 15s.—A number of books have appeared in recent years on this subject, which is evidence of its importance. Kanavel's pioneer work is well known and the present volume represents the most up-to-date exposition of the subject, in which it is urged that " finger clinics " should be instituted and encouraged at all large hospitals. As the dedication implies, the author is in entire accord with Kanavel's teaching, and he adopts all the practical suggestions of that surgeon, giving especial importance to the palmar spaces and their infection. He lays great stress on the use of the sulphonamide drugs in the treatment of streptococcal infection and he gives a timely warning against the use of incisions in cases of lymphangitis. Considerable space is devoted to the restoration of function as being the ultimate aim of all surgical treatment. In describing the use of physio-therapy emphasis is laid on the value of radiant heat and the encouragement of active rather than passive movements. The treatment of fractures is described according to the lines laid down by Böhler and Watson-Jones. The book is well illustrated and will be welcomed as a practical guide both by students and practitioners.

**Surgery of the Hand.** By JOHN HAROLD COUCH, M.B., F.R.C.S. Pp. ix., 147. Illustrated. London : Oxford University Press (Humphrey Milford). 1939. Price 7s.—In the Toronto General Hospital the policy of specialization both in teaching and in practice has been carried very far. For ten years all the surgical staff has made Dr. Couch into an adviser and consultant in hand cases. The Dean, Dr. Gallie, is confident that this policy has been justified by its results. The present book is the outcome of the experience gained in these ten years. It deals, however, with general principles rather than minute details. It is very well illustrated by line drawings, which are models of clearness. It is remarkable that so much practical knowledge can be imparted by such a small book. The economic importance of hand injuries and especially of infection is rightly stressed. Details are given of the suture of tendons and nerves and of the principles of amputation. In regard to infection the general lines of Kanavel's work are followed, but there is no mention of the palmar spaces. We have no doubt the book will be of great value to students and practitioners.

**Introduction to Pharmacology and Therapeutics.** Sixth Edition. By J. A. GUNN, M.D., D.Sc., F.R.C.P. Pp. viii., 242. London : Oxford University Press (Humphrey Milford). 1940. Price 6s.—This is a revised edition of an old and reliable friend. The small volume is in no sense a cram book, but contains a solid foundation for a more detailed study of pharmacology and therapeutics. Dr. Gunn has had wide experience in teaching his subject to medical students, and unlike some pharmacologists gives much help to the students who of necessity must be acquainted also with the principles of therapeutics. The text-book not only contains a fund of information for students, but is also a valuable book of reference. During recent years increased importance has been attached to the functions of the autonomic nervous system, and the author devotes two chapters to matters relating to this system, and gives practical information concerning those drugs which act on one or other of its branches. Two other important chapters deal with depressants of the central nervous system, and here alcohol and other hypnotics are discussed. We are sure that this new edition will find a place in many libraries.

**Second Addendum to the British Pharmacopoeia, 1932.** Pp. ix, 22. London: General Medical Council. 1940.—The Second Addendum to the *British Pharmacopoeia*, 1932, is quite short, containing about fifteen monographs and five appendices. Nearly the whole of the monographs represent the embodiment in the *Pharmacopoeia* of vitamin preparations, including halibut liver oil and 50 per cent. emulsion of cod liver oil. In view of the increasing number of vitamin preparations placed on the market by private firms, each with its own proprietary name and arbitrary standard, the recognition of official standards will be welcome. The Addendum includes some modified formulæ for four ointments, legalizing the use of cotton seed and sesame oils during war conditions, and an improved formula for tannic acid ointment. Tetanus toxoid is also included. The entire book represents the current work of the standing Pharmacopeia Committees of the General Medical Council. The appendices embody tests and assays rendered necessary by the inclusion of the new monographs.

**Third Addendum to the British Pharmacopoeia, 1932.** Pp. viii, 32. London: General Medical Council. 1940.—The Third Addendum is essentially a war product, although in some ways its innovations may possibly mark a milestone in chemical materia medica. The war has prompted the inclusion of a number of synthetic chemicals to take the place of their therapeutic equivalents, largely of foreign origin, which have flooded the advertisement pages of the medical and pharmaceutical press ever since the last war. It might be an excellent outcome if the General Medical Council after the war insisted on the disclosure of formulae, clinical examination and trial of all advertised (including ethically advertised) new remedies introduced, also the prohibition of use of multiple names for the same article. The book is made up of the following components: (1) The synthetic compounds referred to above, together with their alleged therapeutic equivalents; (2) Preparations in the main volume of the *British Pharmacopoeia* in which olive oil can be replaced by arachis and one in which white bees wax can be used instead of benzoated lard; (3) A few new preparations are added, namely, aneurin hydrochloride, vitamin $B_1$, bromethol, injectio leprazoli (injection of cardiazol), and pasta acid tannic. The Addendum as a whole may be regarded as a very useful contribution to the materia medica of the wartime period and later.

[NOTE.—It would require fresh legislation to give the G.M.C. power to do this.—ED.].

